# Effect of the Transient Pharmacological Inhibition of Mapk3/1 Pathway on Ovulation in Mice

**DOI:** 10.1371/journal.pone.0119387

**Published:** 2015-03-24

**Authors:** Dayananda Siddappa, Élaine Beaulieu, Nicolas Gévry, Philippe P. Roux, Vilceu Bordignon, Raj Duggavathi

**Affiliations:** 1 Department of Animal Science, McGill University, Ste-Anne-de-Bellevue, QC H9X 3V9, Canada; 2 Département de Biologie, Université de Sherbrooke, Sherbrooke, QC J1K 2R1, Canada; 3 Institute for Research in Immunology and Cancer, Faculty of Medicine, Université de Montréal, Montreal, QC H3C 3J7, Canada; Clermont Université, FRANCE

## Abstract

Mitogen-activated protein kinase 3/1 (Mapk3/1) pathway is critical for LH signal transduction during ovulation. However, the mechanisms remain incompletely understood. We hypothesized that Mapk pathway regulates ovulation through transcriptional regulation of ovulatory genes. To test this hypothesis we used immature mice superovulated with equine and human chorionic gonadotropins (eCG and hCG) and PD0325901, to inhibit hCG-induced Mapk3/1 activity. Mice received either the inhibitor PD0325901 (25 μg/g, i.p.) or vehicle at 2h before hCG stimulation. Administration of the inhibitor abolished Mapk3/1 phosphorylation in granulosa cells. While vehicle-treated mice ovulated normally, there were no ovulations in inhibitor-treated mice. First, we analyzed gene expression in granulosa cells at 0h, 1h and 4h post-hCG. There was expected hCG-driven increase in mRNA abundance of many ovulation-related genes including *Ptgs2* in vehicle-treated granulosa cells, but not (P<0.05) in inhibitor-treated group. There was also reduced mRNA and protein abundance of the transcription factor, early growth response 1 (Egr1) in inhibitor-treated granulosa cells. We then used GRMO2 cell-line to test if Egr1 is recruited to promoter of *Ptgs2* followed by chromatin immunoprecipitation with either Egr1 or control antibody. Enrichment of the promoter regions in immunoprecipitants of Egr1 antibody indicated that Egr1 binds to the *Ptgs2* promoter. We then knocked down Egr1 expression in mouse primary granulosa cells using siRNA technology. Treatment with *Egr1*-siRNA inhibited Egr1 transcript accumulation, which was associated with reduced expression of *Ptgs2* when compared to control-siRNA treated granulosa cells. These data demonstrate that transient inhibition of LH-stimulated MAPK3/1 activity abrogates ovulation in mice. We conclude that Mapk3/1 regulates ovulation, at least in part, through Egr1 and its target gene, *Ptgs2* in granulosa cells of ovulating follicles in mice.

## Introduction

Ovulation is a multi-gene, multi-step process involving complex signaling pathways, which facilitates synchronization of oocyte maturation and cumulus expansion with that of follicular rupture. It is unequivocal that preovulatory luteinizing hormone (LH) surge initiates these processes through remarkable changes in gene expression program of granulosa cells within ovulating follicles. Some of the important signaling pathways through which LH brings about ovulatory events are cAMP/Protein Kinase A (PKA) pathway, Mitogen-activated protein kinase 3/1 (Mapk3/1; ERK1/2) pathway and phosphatidylinositide 3-kinases (PI3K) pathway [1–4]. A recent study using granulosa-specific *Mapk3/1* knockout (KO) mice [5] provided *in vivo* evidence for the importance of Mapk3/1 signaling in LH signaling during ovulation.

Granulosa cells from *Mapk3/1* KO mice showed altered expression of hundreds of LH regulated genes [5], but which transcription factors act as mediators of their signals have not been completely identified [6]. Many transcription factors including nuclear receptor 5a2 (Nr5a2) [7] (CAAT/enhancer binding protein beta (Cebpb) [6], early growth response-1 (Egr1) [8] and Progesterone receptor (Pgr) [9] are critical LH signaling during ovulation. It was reported that 19% of the LH-driven genes were regulated in granulosa cells of both *Mapk3/1* and *Cebpa/b* conditional KO mice at 4h hCG [6]. This indicates that the rest 81% Mapk3/1-dependent genes are regulated by transcription factors other than Cebpa/b, which are yet to be identified.

While conditional KO model is a powerful tool to study physiological processes in vivo, it is not devoid of limitations. For example expression of the Cre-recombinase may not be active in all cells of interest, therefore, leading to incomplete gene deletion. On the other hand, pharmacologic method of inhibition of a protein’s activity is economical, less time consuming and relatively simple compared to genetic manipulation. Moreover, using pharmacologic method one can inhibit protein activity activity transiently at a precise physiological stage. Limitations of pharmacologic methods include potential “off-target” effects. PD0325901 is specific inhibitor of Mapk-kinase (Map2k; MEK), which abrogates Mapk3/1 activity without cytotoxicity when administered as a single dose of 25μg/g bodyweight in mice [10,11]. More importantly, PD0325901 does not have off-target effects shown by other Map2k inhibitors, U0126 and PD98059 [12]. Therefore, PD0325901 treatment is an excellent alterative method to inhibit Mapk3/1 activity at precise time-points during follicular development.

The aim of our study was to identify novel transcription factors that play an important role downstream of Mapk3/1 signaling in the process of ovulation. We hypothesized that Mapk3/1 pathway regulates ovulation through transcriptional regulation of ovulatory genes. To test this hypothesis we employed an *in vivo* pharmacologic method of inhibition of Mapk3/1 activity with out disrupting the *Mapk3/1* gene expression. Here we report our study exploring the effect of PD0325901 on ovulation in superovulated immature mice.

## Materials and Methods

### Animals and treatments

#### Husbandry

Inbred C57BL/6NCrl mice (Charles River) were housed in standard plastic rodent cages and maintained on a 12-h light/dark cycle with *ad libitum* feed (Teklad-Rodent irradiated Diet, Harlan) and water. The animal use protocol was approved by the Animal Care and Use Committee, McGill University.

#### Ovarian superstimulation

Immature mice (23–25d old) were first treated with equine chorionic gonadotropin (eCG; Sigma Life Sciences; 5 IU i.p.) to stimulate follicle development. Forty-eight hours later, mice were treated with human chorionic gonadotropin (hCG; Sigma Life Sciences; 5 IU i.p.) to induce ovulation and luteinization. In this protocol, the ovulation occurs at 12–14h post-hCG [7,13,14].

#### Inhibition of Mapk3/1 activity

A potent selective Map2k (MEK) inhibitor PD0325901 (Selleckchem) was initially dissolved in DMSO (Fisher Scientific) to prepare a stock solution of 100 μg/μl concentration. A dosing solution of 2.5 μg/μl in 5% DMSO in saline was prepared just before treatment. For inhibition of Mapk3/1 activity, mice were administered with a single dose of PD0325901 (25 μg/g body weight, i.p.) 2h before hCG treatment. PD0325901 will be referred to as Map2k-inibitor from here on for readers’ simplicity. Mice treated with 5% DMSO in saline served as vehicle controls. We determined Mapk3/1 activity by determining the abundance of phosphorylated isoform of Mapk3/1 relative to its total isoform. The dose of Map2k-inhibitor was determined based on a preliminary experiment with doses of 10 and 25 μg/g 2 h before hCG treatment, of which the later dose resulted in consistent inhibition of Mapk3/1 activity.

### Ovulation rate and histology

To study the effect of inhibition of Mapk3/1 activity on ovulation rate, we counted cumulus oocyte complexes (COCs) from both oviducts of Map2k-inhibitor and vehicle treated mice at 18h post-hCG. Ovaries collected at 18h post-hCG from these mice were fixed in 10% neutral buffered formalin at 4°C for 2 days. Paraffin embedded ovaries were cut (4μ thickness) and sections were stained by hematoxylin and eosin for histological observation under Leica DM200 microscope attached with Leica EC3 camera.

### Granulosa cell collection and real-time PCR

Granulosa cells were collected by follicle puncture [15] at 0h, 1h and 4h post-hCG (N = 3 mice/group). Briefly, granulosa cells were collected by follicular puncture using 27G needle. The cell suspension was passed through a cell strainer (BD Falcon, Mississauga, ON, Canada; 40μm) to filter out cumulus–oocyte complexes. Pure populations of mural granulosa cells from both ovaries of each mouse were pooled together. Total RNA was purified from granulosa cells using Picopure RNA isolation kit (Arcturus Biosciences) followed by cDNA synthesis from 500 ng of RNA using iScript kit (Bio-Rad). We analyzed mRNA expression by real-time PCR as previously described [14] using the primers shown in Table 1. Expression data for each gene of interest was normalized to mean expression levels of four reference genes (*B2m*, *Sdha*, *L19 and Gapdh*).

**Table 1:** Primer sequence for real time PCR.

Gene	Forward	Reverse
Adamts1	CATAACAATGCTGCTATGTGCG	TGTCCGGCTGCAACTTCAG
Areg	AGGGGACTACGACTACTCAG	GAAACTTGGCAGTGCATGGA
Egr1	TCGGCTCCTTTCCTCACTCA	CTCATAGGGTTGTTCGCTCGG
Fshr	GTGCTCACCAAGCTTCGAGCTAT	AAGGCCTCAGGGTTGATGTACAG
Has2	TGTGAGAGGTTTCTATGTGTCCT	ACCGTACAGTCCAAATGAGAAGT
Mapk1	CAGGTGTTCGACGTAGGGC	TCTGGTGCTCAAAAGGACTGA
Mapk3	TCCGCCATGAGAATGTTATAGGC	GGTGTTGATAAGCAGATTGG
Nr5a2	TCATGCTGCCCAAAGTGGAGA	TGGTTTTGGACAGTTCGCTT
Pappa	CACAGGCAGAGCATCAGGAAG	TGCTTGCCATGAGGTAACCAG
Pgr	GGTGGAGGTCGTACAAGCAT	CTCATGGGTCACCTGGAGTT
Ptgs2	TGAGCAACTATTCCAAACCAGC	GCACGTAGTCTTCGATCACTATC
Ptgs2-ChIP	CGCAACTCACTGAAGCAGAG	ATGGGGAGAACCTTGCTTTT
Ptx3	CCTGCGATCCTGCTTTGTG	GGTGGGATGAAGTCCATTGTC
Scarb1	TTTGGAGTGGTAGTAAAAAGGGC	TGACATCAGGGACTCAGAGTAG
Star	CCGGGTGGATGGGTCAA	CACCTCTCCCTGCTGGATGTA
Tnfaip6	GGGATTCAAGAACGGGATCTTT	TCAAATTCACATACGGCCTTGG

### Protein extraction and immunoblot analyses

Granulosa cells were collected in Laemmli buffer (Bio-rad) containing DTT (Omnipur), phosphatase and protease inhibitors (G Biosciences), and were boiled at 95°C for five minutes. Protein extracts were resolved by polyacrylamide electrophoresis and transferred to nitrocellulose membranes. After blocking with 5% milk in TBS-T, the membranes were incubated overnight at 4°C with primary antibodies (1:1000) followed by washing with TBS-T (3X 10 min each) and incubation with secondary antibody (1:10000) for 1hour at room temperature. The immunoblotted proteins were detected using Immun-Star Kit and Chemidoc Analyzer (BioRad). Whenever necessary, the membranes blotted with one primary antibody were stripped using stripping buffer (10% SDS, 0.5 M Tris-Hcl, DEPC H2O ml and 2-mercaptoethanol) and re-blotted with another antibody. Antibodies used: MAPK3/1 (#4695), phoshpho-MAPK3/1 (Thr202/Tyr204)(#4376) from Cell Signaling; EGR-1 (#sc-189x) form Santa cruz biotechnology; beta actin (ab8227) and Goat anti-rabbit-IgG (ab6721) from Abcam.

### GRMO2 cell culture and cAMP treatment

Mouse granulosa cell line, GRMO2 cells were cultured in DMEM/F12 (Wisent) supplemented with 5μg/ml insulin, 10μg/ml transferrin, 30pM selenite solution (Wisent), 2% fetal bovine serum (Wisent), penicillin-streptomycin (Wisent) and 3μg/ml BSA (Sigma) at 37°C and 5% CO2 in a humidified incubator [16]. GRMO2 cells were treated with 1mM 8Br-cAMP (Sigma) or vehicle for 4 hours to simulate preovulatory LH treatment. Cells were then harvested after 4h (3–5 wells per treatment) for either gene expression analyses by qPCR detailed above or chromatin immmunoprecipitation described below.

### Chromatin immunoprecipitation

Chromatin immunoprecipitation (ChIP) assays were performed as described previously [17]. Briefly, cells were cross-linked for 10 min at room temperature with 1% formaldehyde in PBS. Cells were then washed in PBS, resuspended in 200 μl of ChIP lysis buffer [1% SDS, 10 mm EDTA, 50 mm Tris-HCl (pH 8.0), and protease inhibitors], and sonicated. The chromatin solution was diluted 10-fold in ChIP dilution buffer [0.01% SDS, 1.1% Triton X-100, 1.2 mm EDTA, 16.7 mm Tris (pH 8.1), 16.7 mm NaCl, and protease inhibitors]; 5% of the lysate was used for purification of input DNA. Each sample was precleared by incubating with 2 μg of salmon sperm DNA/protein A-agarose 50% gel slurry (Roche Diagnostics) for 2 h at 4°C. Two to 4 μg of the Egr1 antibody or rabbit IgG was added and immunoprecipitated at 4°C overnight. The immunoprecipitant was collected using salmon sperm DNA/protein A-agarose and washed sequentially with the following buffers: low-salt wash buffer [0.1% SDS, 1% Triton X-100, 2 mm EDTA, 20 mm Tri-HCl (pH 8.1), and 150 mm NaCl], high-salt wash buffer [0.1% SDS, 1% Triton X-100, 2 mm EDTA, 20 mm Tris-HCl (pH 8.1), and LiCl wash buffer [0.25 m LiCl, 1% Nonidet P-40, 1% sodium deoxycholate, 1 mm EDTA, and 10 mm Tris-HCl (pH 8.1)], and Tris-EDTA buffer [10 mm Tris-HCl (pH 8.0) and 1 mm EDTA]. DNA-protein cross-links were reversed by incubation at 65°C overnight followed by proteinase K treatment. DNA was purified with Qiaquik PCR purification column (QIAGEN).

### Primary granulosa cell culture and siRNA-induced Egr1 gene knockdown

Granulosa cells from immature mice were collected, as described above, at 40h after eCG administration under aseptic conditions. Granulosa cell suspension was centrifuged at 1000g for 5 min at 37°C and pellet was re-suspended in electroporation medium (DMEM/F12 glutamax, Gibco 10565–018). Following cell counting using a hemocytometer, granulosa cells were diluted to a final concentration of 0.2 X10^6^ cells in 10μl of DMEM/F12 media. Homogenous granulosa cell suspension was split into three parts. Each part was then mixed with either medium or control siRNA (at 20nM conc, Dharmacon, D-001210–05–05) or Egr1 siRNA smart pool (20nM, Dharmacon, M-040286–01). Electroporation was done using the Neon Transfection System (MPK 1096–772) and MBI microporator (Digital Bio) with the settings of 1000 volts, 30 millisecond and 3 pulses. Electroporated granulosa cells were plated in 24-well tissue culture plate (Sarstedt; 0.2 X10^6^ cell/well) containing pre-warmed medium and incubated for 6 h at 37°C and 5% CO_2_. At the end of 6h incubation period, granulosa cells were treated with either forskolin (Fo; 10μM, Sigma, P3917) and phorbol-12-myristate (PMA; 20μM, Sigma, P1585) or medium for 4 h. Combined treatment with Fo and PMA (Fo+PMA) was used to mimic LH or hCG treatment *in vivo*. Following this treatment period, granulosa cells were harvested for either transcript or protein analysis.

### Statistical analysis

For data involving time and treatment, we used SigmaPlot 12.3 software two-way ANOVA followed by Holm-Sidak analysis for multiple comparisons. All data are represented as mean ± SEM and p < 0.05 was considered statistically significant.

## Results

### Map2k-inhibitor abolishes LH induced Mapk3/1 activity in granulosa cells

**Fig 1:**
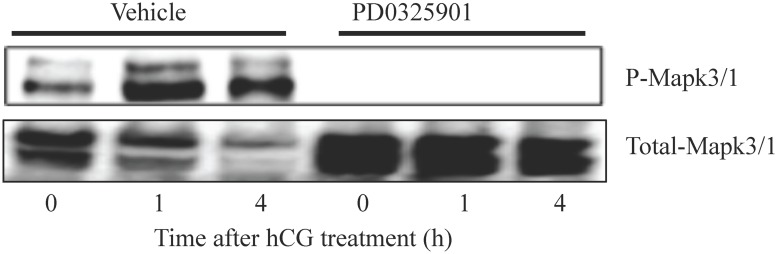
Transient inhibition of hCG induced Mapk 3/1 activity by Map2k inhibitor treatment during ovarian superstimulation protocol. Granulosa cells collected by follicular puncture from mice treated with PD0325901 showed absence of phosphorylation of Mapk3/1 at Thr302/Tyr204 compared to vehicle treated mice (n = 3 mice/group/time point).

We first determined whether Map2k-inhibitor could be used as pharmacological inhibitor of granulosa cell Mapk3/1 activity during hCG-induced preovulatory follicle maturation in mice. Treatment with the inhbitor at 2h before hCG stimulation dramatically reduced the relative abundance of phospho-Mapk3/1 in granulosa cells of ovulating follicles compared to vehicle treatment (Fig. 1).

### Inhibition of Mapk3/1 activity in preovulatory follicles abrogates ovulation

**Fig 2:**
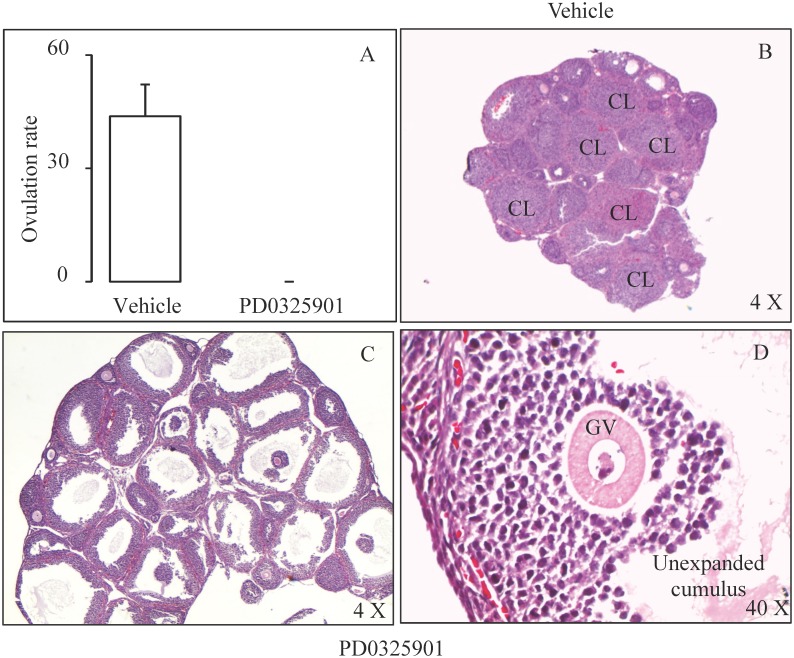
Inhibition of hCG induced cumulus expansion, oocyte maturation and follicular rupture due to Map2k inhibitor treatment. Treatment of immature mice with single dose of 25μg/g of PD0325901 resulted in anovulation compared to vehicle treated mice (A) during superovulation (n = 5 mice/ group). This anovulation as evidenced by trapped GV stage oocytes with compact cumulus cells (C&D) inside the preovulatory follicles at 18h hCG (n = 5 mice/ group). Vehicle treated mice showed well-developed corpus luteum (B) evidencing follicular rupture, ovulation and luteinization. CL—corpus luteum; GV—germinal vesicle.

Having established the experimental paradigm to efficiently inhibit hCG-induced Mapk3/1 activity using a pharmacological inhibitor, we asked whether this inhibition would have any effect on the ovulatory process. We found that Map2k-inhibitor treatment resulted in complete anovulation while vehicle treated mice ovulated normally (Fig. 2A). Histology of ovaries of control mice at 18h post-hCG showed numerous well-developed corpora lutea attesting to normal follicular rupture and luteinization (Fig. 2B). In contrast, ovaries of Map2k-inhibitor treated mice showed non-luteinized un-ruptured follicles with trapped oocytes. The follicles did not show any signs of luteinization and oocytes were in germinal vesicle stage surrounded by unexpanded cumulus (Fig. 2C and D).

### Pharmacological inhibition of Mapk3/1 activity does not inhibit global transcription in granulosa cells, nor does it cause toxicity in mice

**Fig 3:**
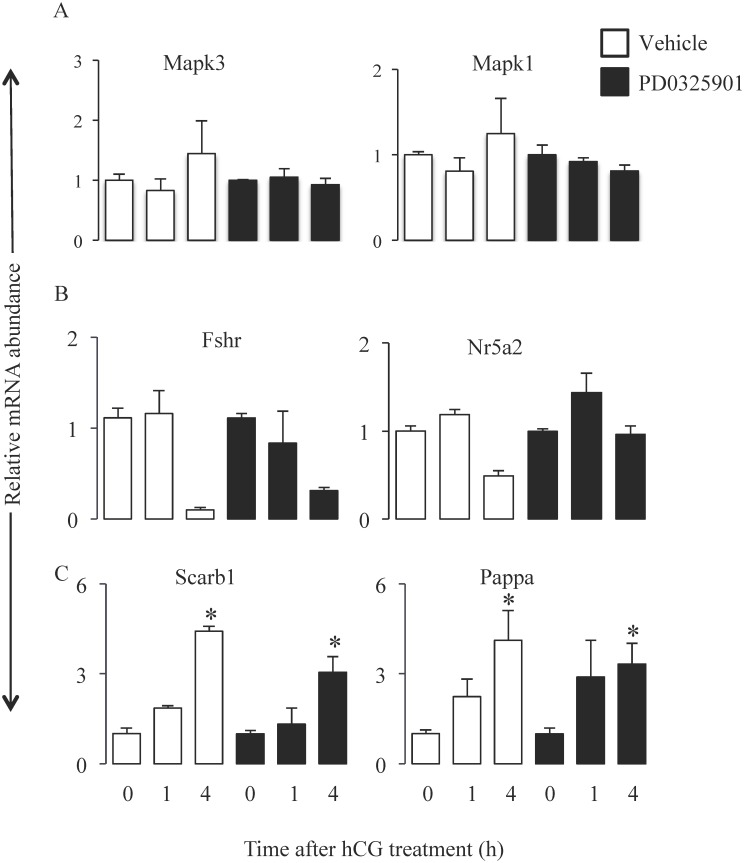
Map2k inhibitor treatment does not shutdown the global transcription in granulosa cells. Treatment of immature mice with single dose of 25μg/g of PD0325901 did not alter relative mRNA abundance of Mapk1 and Mapk3 (A). Expression of known hCG regulated genes like *Fshr* and *Nr5a2* (B) as well as hCG induced genes like *Scarb1* and *Pappa* (C) were also not altered in PD0325901 treated mice (n = 3mice/group/time point).

To investigate the mechanisms underpinning anovulation in the absence of Mapk3/1 activity, we undertook molecular phenotyping of granulosa cells collected at 0, 1 and 4h post-hCG in control and Map2k-inhibitor treated mice. Even though Map2k-inhibitor completely abolished Mapk3/1 activity in granulosa cells, it did not alter the relative mRNA abundance of *Mapk3/1* in granulosa cells (Fig. 3A). Expression pattern of *Fshr* and *Nr5a2* from 0h to 4h post-hCG was similar in granulosa cells of both control and Map2k-inhibitor treated mice (Fig. 3B). Interestingly, relative mRNA abundance of *Scarb1* and *Pappa* increased from 0h to 4h post-hCG in granulosa cells of both groups of mice (Fig. 3C). Further administration of vehicle or Map2k-inhibitor to mice did not produce any general signs of toxicity. These mice were active and appeared healthy upon physical examination. Paraffin sections of the liver and kidney of vehicle or Map2k-inhibitor treated mice were similar histologically and did not show any obvious signs of toxicity at 18h post-hCG (data not shown). Histologically ovary from Vehicle or Map2k-inhibitor treated mice did not showed any signs of toxicity, except absence of ovulation.

### Expression of hCG-induced ovulatory genes is decreased in granulosa cells lacking Mapk3/1 activity

**Fig 4:**
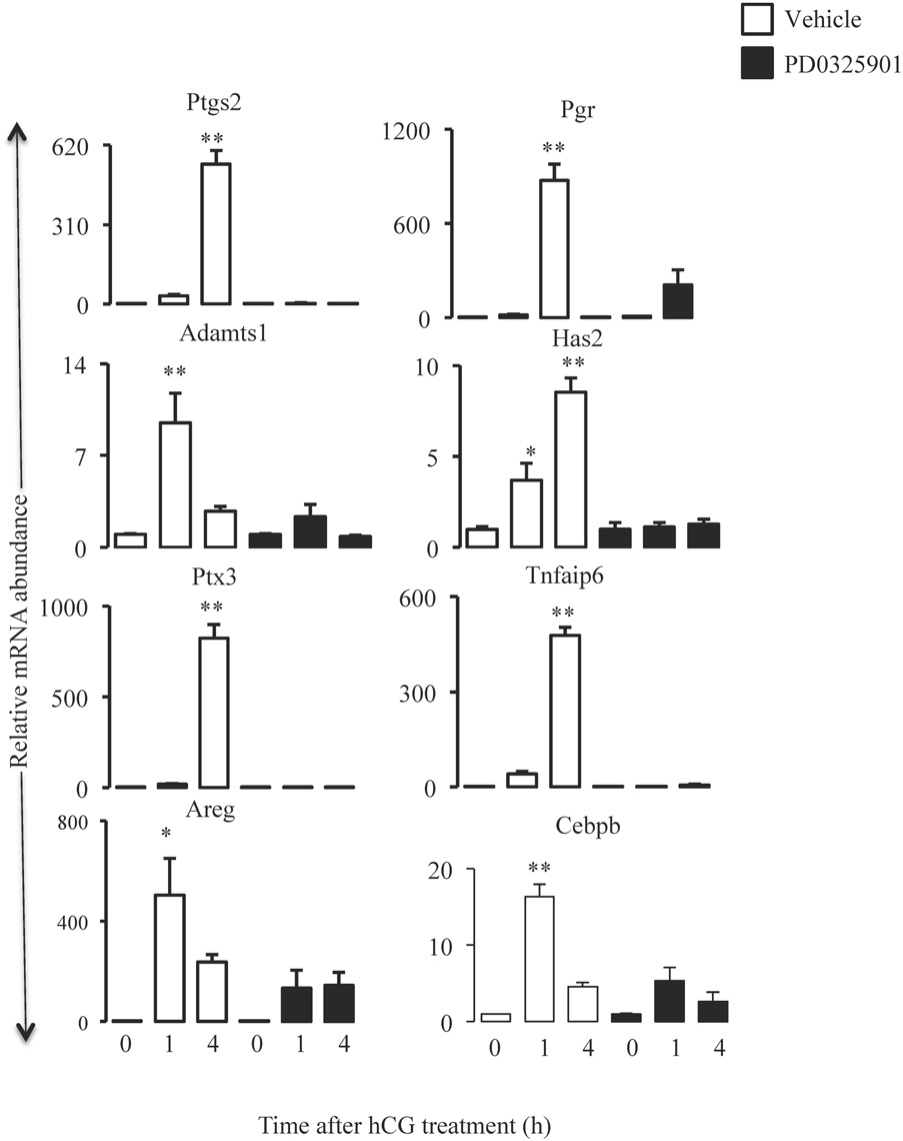
Map2k inhibitor treatment reduces the expression of hCG induced ovulatory genes. Treatment of immature mice with single dose of 25μg/g of PD0325901 inhibited the expression of hCG-induced genes involved in follicular rupture like *Ptgs2*, *Pgr*, *Adamts1*; genes involved in cumulus expansion like *Has2*, *Ptx3*, *Tnfaip6*; gene involved in oocyte meiotic maturation like Areg and luteinization like *Cebpb* (n = 3mice/group/time point). Mapk3/1 regulated LH induced genes like *Ptgs2*, *Ptx3 and Tnfaip6* were not regulated by Cebpb.

Expression patterns of the genes implicated in the process of ovulation in vehicle and Map2k-inhibitor treated mice are shown in Fig. 4. One of the most important genes induced in granulosa cells by preovulatory LH surge is the prostaglandin synthase 2 (*Ptgs2*), which is critical for both follicular rupture and cumulus expansion [18–20]. At 4h post-hCG a 545-fold increase in *Ptgs2* mRNA was observed in granulosa cells of vehicle treated mice, but such a dramatic increase was absent (P<0.001) in granulosa cells of Map2k-inhibitor treated mice. The two other genes that play an important role in follicular rupture are progesterone receptor (*Pgr*) and A disintegrin and metalloproteinase with thrombospondin motifs 1 (*Adamts1)*. In granulosa cells of vehicle treated mice *Pgr* mRNA was induced by 876-fold at 4h post-hCG compared to 0h hCG. Such a remarkable induction was completely absent in Map2k-inhibitor treated mice (P<0.001). Likewise, in Map2k-inhibitor treated mice, lack of Mapk3/1 activity abolished (P<0.001) a 9-fold increase in *Adamts1* mRNA observed in granulosa cells of vehicle treated mice. Apart from Ptgs2, genes that mediate cumulus expansion include Hyaluronan synthase 2 (*Has2*), TNFα-induced protein 6 (*Tnfaip6*) and pentraxin 3 (*Ptx3*). In vehicle treated mice increase in *Has2* mRNA abundance was 8-fold at 4h post-hCG. Map2k-inhibitor treatment abrogated such an increase in the mRNA expression of *Has2* at 4h hCG (P<0.001). Similarly, 822-fold increase in *Ptx3* and 476-fold increase in *Tnfaip6* mRNA expressions seen in granulosa cells of vehicle treated mice was absent (P<0.001) in Map2k-inhibitor treated mice. Epidermal growth Factor (EGF)-like growth factors namely *Areg* (amphiregulin), *Ereg* (Epiregulin) and *Btc* (Betacellulin) have been implicated in oocyte meiotic maturation [3], of which we examined the expression pattern of *Areg*. Expression of *Areg* mRNA was highest at 1h hCG in vehicle treated granulosa cells, while its induction was reduced (P<0.05) by 50% in Map2k-inhibitor treated granulosa cells. *Cebpb* has been implicated in regulation of the terminal differentiation of granulosa cells during ovulation[6]. In vehicle treated mice granulosa cells *Cebpb* mRNA expression was highest at 1h hCG and it was downregulated (P<0.001) in Map2k-inhibitor treated mice.

### Inhibition of Mapk3/1 activity reduces the expression of transcription factor Egr1

**Fig 5:**
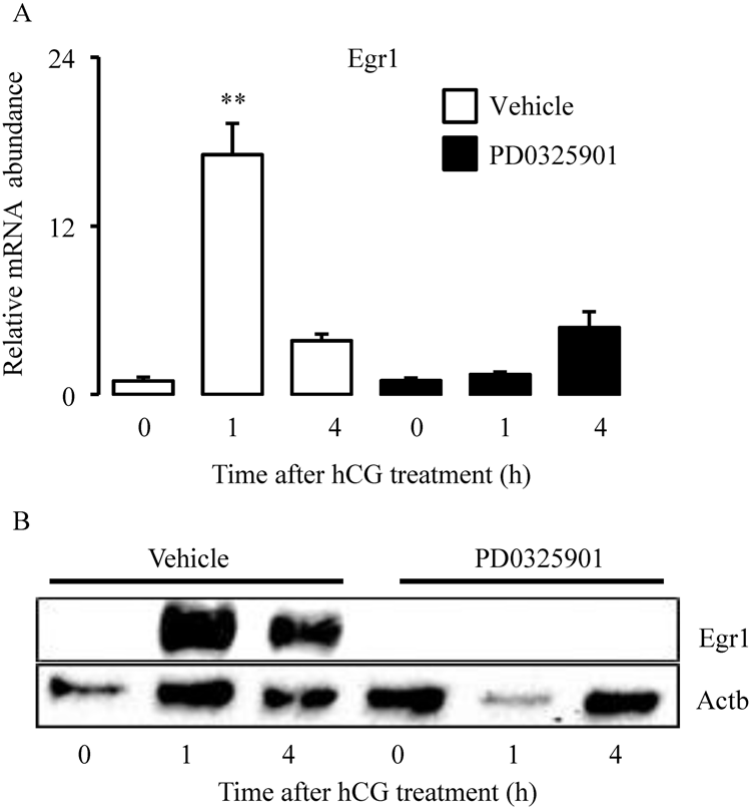
Transient inhibition of Mapk3/1 reduces transcription and translation of transcription factor Egr1. A) Treatment of immature mice with single dose of 25μg/g of PD0325901 inhibited the expression of hCG-induced expression of *Egr1* 1h and 4h hCG. B) Abundance of Egr1 protein in granulosa cells of the inhibitor and vehicle treated mice. Actb was used as loading control.

Of the major transcription factors involved in ovulation mentioned in the introduction, Nr5a2 was not affected by Mapk3/1 inhibition (Fig. 3); *Pgr* was not expressed at 1h (Fig. 4), and Cebpb appears to be dispensable for early events of preovulatory LH signaling [6]. Therefore, we asked the question as to which transcription factor could mediate Mapk3/1 regulated transcription of LH-induced genes like Ptgs2. As it has been well established that LH/hCG induces Egr1 expression in granulosa cells during ovulation [21,22], we hypothesized that this Mapk3/1 activity would be indispensible for the expression of the transcription factor, early growth response-factor 1 (Egr1). Indeed, inhibition of Mapk3/1 activity abolished (P<0.001) LH-induced mRNA expression of *Egr1* at 1h post-hCG as compared to vehicle treatment (Fig. 5A). Confirming these mRNA data, relative abundance of Egr1 protein was remarkably absent in granulosa cells lacking Mapk3/1 activity at 1h and 4h post-hCG as compared to control granulosa cells (Fig. 5B).

### Egr1 binds to promoter regions of *Ptgs2*

**Fig 6:**
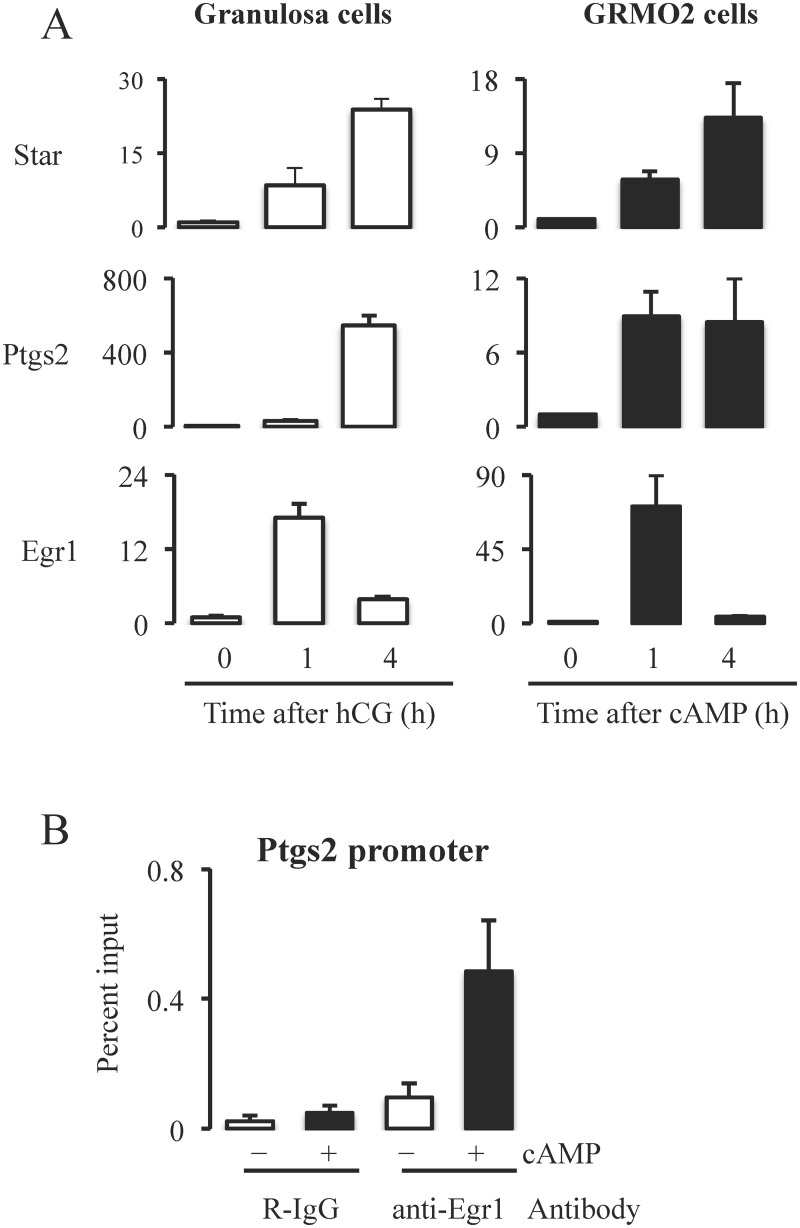
A) Comparison of expression profile of ovulatory genes in granulosa cells from hCG treated mice and cAMP treated GRMO2 cells. Expression profiles of ovulatory genes like *Star*, *Ptgs2* and *Egr1* were similar at 0h, 1h and 4h after hCG or cAMP treatment in granulosa cells or cultured GRMO2 cells respectively. B) Binding of Egr1 transcription factor to Ptgs2 promoter region (-159 to -33 relative to the transcription start site). Chromatin immunoprecipitation and qPCR using 4h cAMP treated GRMO2 cells showed the enrichment of promoter region containing Egr1 binding site (identified by bioinformatic analysis) in immunoprecipitants of Egr1 antibody indicating that Egr1 binds to Ptgs2 promoter. Rabbit IgG was used as a control antibody.

In light of the data describe above, the obvious question was whether Egr1 acts as a downstream effector of Mapk3/1 for induction of the ovulatory genes such as *Ptgs2*. To address this question, we performed ChIP analysis using Egr-1specific antibody. Because ChIP assays require large number of cells, we used a mouse granulosa cell line, GRMO2 cells. First, we characterized gene expression pattern of GRMO2 cells in response to cAMP. GRMO2 cells stimulated with cAMP and granulosa cells from mice treated with hCG showed similar relative expression pattern genes like *Star*, *Ptgs2* and *Egr1* (Fig. 6A). To determine whether Egr1 binds to the promoters of ovulatory genes, we performed ChIP and qPCR using cAMP stimulated GRMO2 cells. Immunoprecipitation with Egr1-specific antibody showed that cAMP treatment of GRMO2 cells for 4 h increased the enrichment of the promoter of *Ptgs2* (region-159 to -33 relative to the transcription start site) compared to GRMO2 cells that were not treated with cAMP (Fig. 6B). However, there was no significant increase in amplification of the regions in GRMO2 cells with or with out cAMP treatment when immunprecipitation was done using rabbit IgG. These data clearly indicated that Egr1 is recruited to the promoter region of *Ptgs2* in response to cAMP treatment that is analogous to LH stimulation.

### Knockdown of Egr1 reduces *Ptgs2* transcript abundance

**Fig 7:**
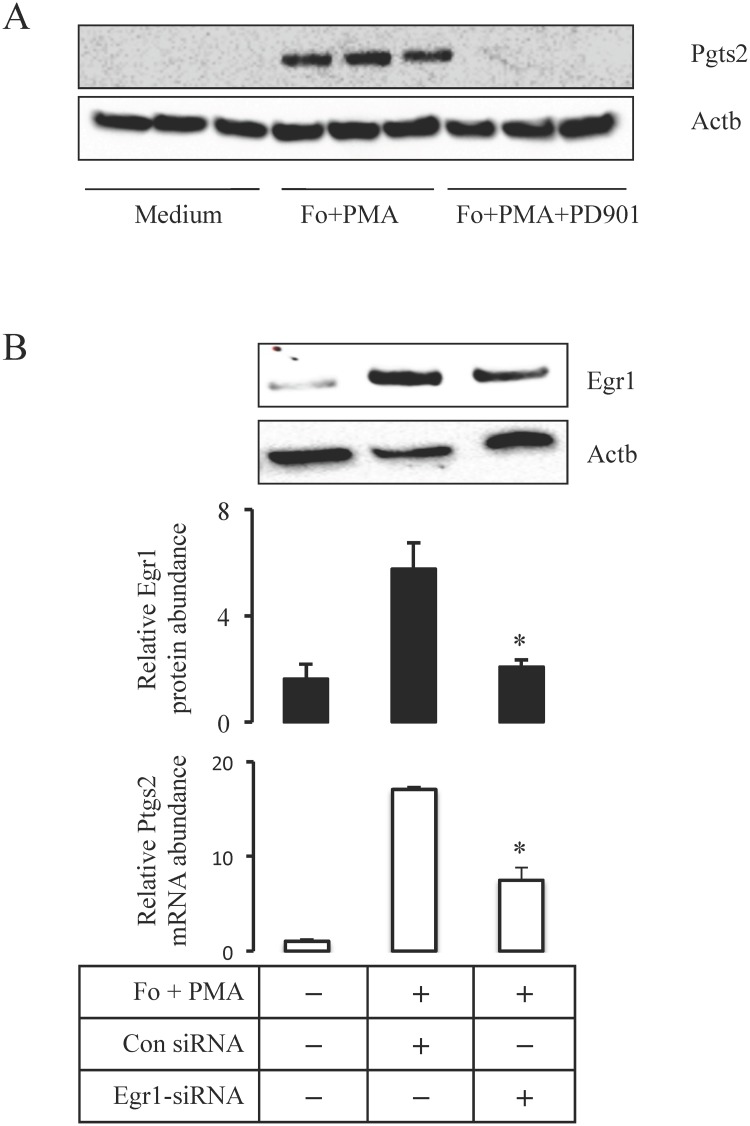
Inhibition of Mapk3/1 activity and knockdown of Egr1 results in reduced induction of Ptgs2 in primary granulosa cells in vitro. A) Treatment with PD0325901 decreased Fo+PMA induced increases in the abundance of Ptgs2 protein relative to vehicle treated primary granulosa cells. B) Pre-treatment with Egr1-siRNA inhibited Fo+PMA induced expression of Egr1 when compared to control siRNA treatment in primary granulosa cells in vitro. This was associated with reduced abundance of Pgts2 transcript in Egr1-siRNA treated cells. Fo—forskolin; PMA—phorbol-12-myristate.

To further demonstrate the importance of Egr1 for *Ptgs2* expression, we used siRNA to knockdown Egr1 expression in mouse primary granulosa cells. Treatment with forskolin and PMA (Fo+PMA) dramatically induced the expression of ovulatory genes including *Egr1* and *Ptgs2* when compared to medium-treated granulosa cells (data not shown). Immunoblot assays revealed that inhibition of Mapk3/1 activity using PD0325901 abolished Ptgs2 induction by treatment with Fo+PMA (Fig. 7A). Treatment of primary granulosa cells with *Egr1*-siRNA reduced Egr1 mRNA and protein abundance compared to levels of control siRNA treated cells (Fig. 7B). Relative mRNA levels of Ptgs2 were 55% lower *Egr1*-siRNA treated granulosa cells as compared to control siRNA treated cells (Fig. 7B).

## Discussion

In the present study we show that transient pharmacological inhibition of Mapk3/1 activity using a single dose of the Map2k inhibitor, PD0325901 during preovulatory follicle maturation resulted in anovulation. This phenotype is similar to the one observed in granulosa-specific *Mapk3/1* KO mice [5]. Our results confirm that this anovulatory phenotype is due to downregulation of numerous genes involved in ovulation. The novel aspect this study is that we have identified the Egr1 as the downstream transcription factor that mediates transcription of genes involved in follicular rupture namely, *Ptgs2* and *Adamts1*.

Ovulation is a process of release of oocyte through rupture of preovulatory follicles[13], regulated by the preovulatory LH-surge. This complex process involves a remarkable gene expression program initiated by LH through multiple signaling pathways and numerous transcription factors. Mapk3/1 are highly conserved Ser/Thr kinases, which regulate cellar proliferation and differentiation [23] by modulating transcription, translation and post translational modifications of their targets [24]. A recent study [5] demonstrated, using granulosa-specific *Mapk3/1*-KO mice, that Mapk3/1 are critical for LH signaling in granulosa cells during ovulation. That study showed Cebpb as a downstream transcription factor responsible for several Mapk3/1 target genes.

Our results showed that transient inhibition of Mapk3/1 activity using single dose of Map2k-inhibitor completely inhibited ovulation in superovulated mice, a phenotype seen in granulosa-specific *Mapk3/1* KO mice [5]. Histology of ovary from inhibitor-treated mice showed disruption of processes of follicular rupture, oocyte maturation, cumulus expansion and luteinization. These phenotypes did not appear to be caused by potential cytotoxic effects of Map2k-inhibitor, as Mapk3/1 were normally expressed in granulosa cells of inhibitor treated mice. The high-density lipoprotein receptor Scarb1 is induced by LH in granulosa cells of mice [7,25] and monkeys [26]. Reduced ovulation rate was observed in *Pappa* KO mice [27], indicating the Pappa may be important for ovulation. Given that both Scarb1 and Pappa, were normally induced in Map2k-inhibitor treated mice, anovulation in these mice does not appear to be due to global abrogation of LH signaling. Indeed, it was previously reported that PD0325901 treatment at 25 ug/g dose, similar to the dose used in this study, did not have any toxic effects [10,11]. Therefore, it is possible to conclude that inhibition of Mapk3/1 activity in granulosa cells resulted in anovulation through inhibition of a specific set of gene involved in the ovulatory processes.

Genes that are induced by hCG and implicated in ovulatory process were remarkably downregulated in granulosa cells of Map2k-inhibitor treated mice. Similar downregulation of multiple genes including, *Ptgs2*, *Pgr* and *Has2* was reported in granulosa-specific *Mapk3/1* KO mice [5]. Ptgs2 appears to play a pleiotropic role during ovulation as ovaries of *Ptgs2* null mice presented absence of CLs, compromised cumulus expansion but normal follicle development with oocyte maturation [18]. On the other hand, Adamts1 and Pgr have been implicated mainly in follicular rupture as mice lacking *Adamts1* [28] or *Pgr* [9] showed ovulatory defects with normal cumulus expansion and luteinization. Even pharmacological inhibition of Pgr using RU486 significantly decreased the number of ovulations [29]. Thus in light of theses reports, our data of lack on LH-induced expression of *Ptgs2*, *Adamts1* and *Pgr* as a result of inhibition of Mapk3/1 activity indicate that Mapk3/1 regulate follicular rupture through transcriptional regulation of the aforementioned genes in granulosa cells.

In the present study Map2k-inhibitor treatment downregulated hCG-induced expression of *Has2*, *Tnfaip6* and *Ptx3* suggesting that Mapk3/1 play a critical role in cumulus expansion through transcriptional regulation of the genes involved in the process. Likewise, granulosa-specific *Mapk3/1* KO mice also showed down regulated cumulus expansion after hCG stimulation. This conclusion is well supported by observations that cumulus expansion is severely compromised in mice null for *Tnfaip6* [30], Ptx3 [31] and *Has2* [32]. Further, inhibition of Mapk3/1 downregulated hCG-induced expression of *Areg* and *Btc*, which are important for cumulus expansion and oocyte meiotic maturation. Indeed, pharmacologic inhibition of Mapk3/1 using U0126 reduced the expression of LH-induced *Areg* and *Ereg* in luteinized human granulosa cells [1]. Also, U0126 treatment inhibited oocyte maturation in cultured large antral follicles [33]. Overall, our mRNA expression data confirm the importance of Mapk3/1 activity in the transcriptional regulation of ovulation-related genes in granulosa cells of ovulating follicles. Therefore, it is important to determine the transcription factors that act as downstream effectors of Mapk3/1 signaling in order to decipher the molecular mechanisms of the regulation of ovulation by this pathway.

Granulosa-specific *Mapk3/1* KO mice showed decreased expression of Cebpb, which was proposed to be one of the downstream effectors of Mapk3/1 signaling in regulating ovulation [5]. It was further demonstrated that granulosa-specific deletion of *Cebpa/b* genes resulted in complete anovulation [6], confirming the mediatory role of these transcription factors in Mapk3/1 signaling. However, this study also revealed that Cebpa/b accounted for 19% of Mapk3/1-regulated genes during early hours of preovulatory differentiation. Most importantly, LH-induced genes like *Areg*, *Ereg*, *Ptgs2*, *Tnfaip6* and *Ptex3* were unaffected in the absence of Cebpa/b in granulosa cells [6]. We also observed downregulation of *Cebpb* expression, which could not explain the dramatic perturbation of the ovulatory gene expression in Map2k-inhibitor treated mice. Therefore, our results along with the genetic studies above clearly demonstrate that Mapk3/1 signaling is complex and may involve several other transcription factors as downstream effectors to regulate gene expression program during ovulation.

Our obvious choice was the immediate early transcription factor, Egr1, which is rapidly and transiently induced by growth factors in many cell types. Our interest in Egr1 was based on several reports. Mice null for *Egr1* were infertile and their ovaries showed follicles of all stages but not corpus luteum [8,34], indicating that it may play a significant role in ovulation. Multiple signaling pathways including Mapk, protein kinase A and C signaling pathways are known to induce *Egr1* expression [35]. Most importantly, LH/hCG can transiently induce *Egr1* exclusively in granulosa cells preovulatory follicles mediating proliferation and/or differentiation during follicular growth, ovulation and luteinization [21,22]. In line with our hypothesis, inhibition of Mapk3/1 activity resulted in remarkable downregulation of hCG-induced Egr1 mRNA and protein. These results clearly indicate that Mapk3/1 activity is indispensable for LH-induced Egr1 expression in granulosa cells.

Several lines of evidence show that Mapk pathway is critical for *Egr1* expression in multiple cell types. Inhibition of Mapk3/1 pathway by Map2k inhibitor PD98059, but not Mapk14 or PI3K pathway, decreased hypoxia induced Egr1 expression in mouse and human alveolar epithelial cells [36]. In bovine luteal cells PGF_2α_ treatment induced *EGR1* through Mapk3/1 pathway and PD098059 challenge inhibited this *EGR1* induction [37]. In rat granulosa cells hCG-induced Egr1 expression was co-regulated by Mapk3/1, cAMP response element binding protein (Creb) and Egr1 itself [21]. However, Creb activity was not altered in granulosa cells null for *Mapk3/1* [5]. Similarly, inhibition of Mapk3/1 activity in the present study showed complete downregulation of Egr1 despite unaltered *Creb* expression (data not shown). Taken together, these data suggest that Mapk3/1 signaling may be more critical than the classical cAMP/Creb pathway for LH-induced Egr1 expression in granulosa cells. Further it has been shown that Egr1 expression is regulated by the transcription factor, serum response factor (Srf) in multiple cell types including granulosa cells [28], gonadotropes [38] and endothelial cells [39]. Since Srf is a known target of Mapk3/1 [40], it is possible to speculate from our data that hCG-induced Egr1 expression in granulosa cells is requires Mapk3/1 kinase activity and the transcription factor Srf during ovulation.

To further link Mapk3/1 dependent Egr1 expression to genes important for follicle rupture, we hypothesized that Egr1 regulates LH-induced transcription of Ptgs2. To test this hypothesis we first used cAMP primed GRMO2 cells. GRMO2 cells have been previously used [16] to study LH-stimulated events in granulosa cells. Systematic analyses revealed that they faithfully recapitulate LH-induced gene expression program in response to cAMP challenge. ChIP analyses using Egr1 antibody clearly demonstrated that Egr1 binds to the promoter of *Ptgs2*. Inhibition of Mapk3/1 activity in primary mouse granulosa cells in vitro resulted in reduced expression of Ptgs2 confirming that Mak3/1 activity is indispensable for Ptgs2 induction during ovulation. Finally, inhibition of Egr1 induction using siRNA technology resulted in reduced expression of Ptgs2 in primary granulosa cells, further confirming that Egr1 regulates Ptgs2 expression in granulosa cells. Our results are further supported by a previous study, which showed that overexpression of EGR1 in bovine granulosa cells increased expression of *PTGS2* expression [41]. Even though *Egr1* KO mice are known to be infertile along with multiple ovarian defects [8], Egr1 target genes have not been explored. With our study demonstrating regulation of Ptgs2 expression by Egr1, it would be very interesting to explore these genes in granulosa cells *Egr1* KO mice.

**Fig 8:**
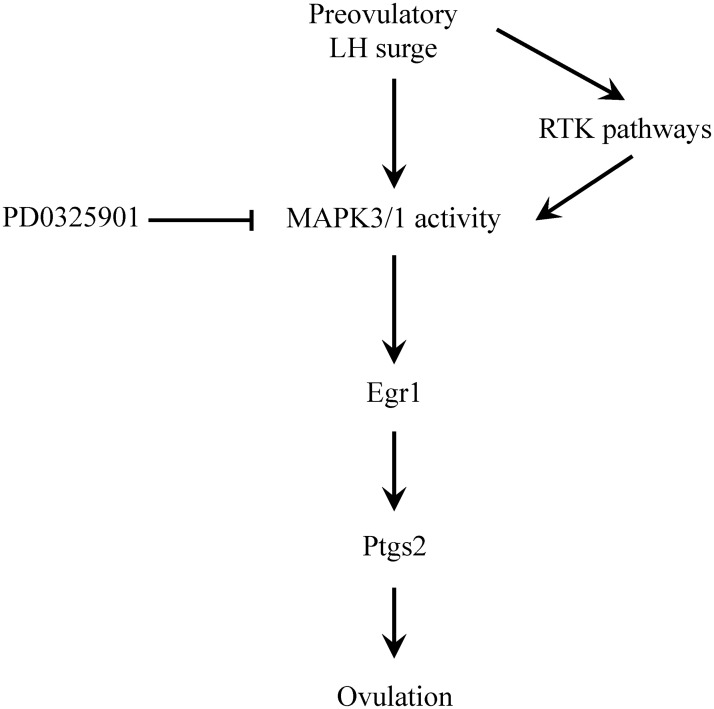
Transcriptional regulation of ovulatory genes by Mapk3/1 through Egr1 transcription factor. Preovulatory LH surge induced Mapk3/1 activity regulates transcription of ovulatory genes such as *Ptgs2* trough transcription factor Egr1. Inhibition of this LH-induced Mapk3/1 activity by Map2k inhibitor PD0325901 results in anovulation.

In conclusion hCG induced Mapk3/1 activity during preovulatory follicle maturation regulates transcription of the genes involved in the process of cumulus expansion, oocyte maturation and follicular rupture. These LH-induced processes are severely compromised in response to a single dose of Map2k-inhibitor PD0325901. Our proposed mechanism for the role of Mapk3/1 is shown in Fig. 8. It is very well established that LH along with modulatory effects of EGF-like growth factors and their tyrosine kinase receptors induces phosphorylation of Mapk3/1. Our results here propose that Egr1 is one of the Mapk3/1 regulated transcription factors that transactivates its target genes involved in follicular rupture such as *Ptgs2*. Finally, several Map2k-inhibitors are under advanced clinical trial for anti-cancer therapy. Since our data here demonstrate that single dose of PD0325901 has an overwhelming inhibitory effect on ovulation in superovulated mice. These dramatic results warrant further detailed studies to test the effect of such inhibitors on ovarian function.
